# Long-Lasting Effects of Changes in Daily Routine during the Pandemic-Related Lockdown on Preschoolers’ Language and Emotional–Behavioral Development: A Moderation Analysis

**DOI:** 10.3390/children10040656

**Published:** 2023-03-30

**Authors:** Valentina Lampis, Sara Mascheretti, Chiara Cantiani, Valentina Riva, Maria Luisa Lorusso, Serena Lecce, Massimo Molteni, Alessandro Antonietti, Marisa Giorgetti

**Affiliations:** 1 Department of Brain and Behavioral Sciences, University of Pavia, 27100 Pavia, PV, Italy; 2 Child Psychopathology Unit, Scientific Institute, IRCCS Eugenio Medea, 23842 Bosisio Parini, LC, Italy; 3 Department of Psychology, Catholic University of the Sacred Heart, 20123 Milan, MI, Italy

**Keywords:** environmental stimulation, COVID-19 pandemic, language skills, emotional–behavioral profiles, moderation, risk/protective factors

## Abstract

The quantity and quality of environmental stimuli and contexts are crucial for children’s development. Following the outbreak of SARS-CoV-2 (COVID-19), restrictive measures have been implemented, constraining children’s social lives and changing their daily routines. To date, there is a lack of research assessing the long-lasting impacts that these changes have had on children’s language and emotional–behavioral development. In a large sample of preschoolers (N = 677), we investigated (a) the long-lasting effects of changes in family and social life and in daily activities over the first Italian nationwide COVID-19-pandemic-related lockdown upon children’s linguistic and emotional–behavioral profiles and (b) how children’s demographic variables and lifelong family characteristics moderated these associations within a multiple-moderator framework. Our findings showed a relationship between the time spent watching TV/playing video games and affective problems that was moderated by the number of siblings. Our findings showed that children who could be at high risk in more normal circumstances, such as only children, have been particularly harmed. Therefore, assessing the long-term effects of lockdown-related measures and how these could have been moderated by potential risk/protective factors added significant information to the existing literature.

## 1. Introduction

The variety of environmental inputs and experiential contexts and the quality of interpersonal stimuli are crucial for the development of children’s cognitive, linguistic, and relational skills and for their psychological wellbeing [1]. Interactions with others are an integral part of a person’s daily life from the moment of birth onward. In addition to being born with a set of predispositions with which to navigate the social world, children’s early years of life represent a “window of opportunity” during which the context of interaction with other humans is critical for optimal brain development and the blossoming of social and communicative skills [2]. A home environment usually represents a privileged context wherein children can be exposed to several enriched experiences [3] and playful and educational materials suitable for their development [4,5]. In addition, the presence of a caregiver allows children to interact with a partner who behaves in a coherent and reassuring way [6] and involves them in meaningful linguistic exchanges [7,8]. Accordingly, several findings suggest that the quality of a child’s home environment impacts their cognitive and socio-emotional development in middle childhood [9]. Similarly, early care and education have considerable short- and long-term effects on children’s cognitive skills (e.g., attention, memory, problem solving, and language) and social-emotional development [10,11]. Both the amount of exposure and the quality of the instructional and social transactions that form toddlers’ early care and education experiences affect the trajectories they will follow when they encounter formal schooling and progress into their middle-childhood years [12].

The outbreak of SARS-CoV-2 (hereafter referred to as COVID-19) has posed a significant threat to the world. Following concern regarding the increasing severity and number of cases the disease along with the declaration of a pandemic status by the World Health Organization [13], governments worldwide took drastic measures and imposed lockdowns during the pandemic to reduce the spread of COVID-19 [14]. Italy was the first European country to be directly impacted by the pandemic [15]. Within the country, Lombardy was the first Italian region to be affected; subsequently, the entire Northern part of Italy recorded a high number of cases [16]. On 8 March (lasting until 11 June) [17], the Italian Government began implementing progressively more restrictive measures, culminating in the first nationwide lockdown, which encompassed isolation, contact restrictions, the closure of schools and kindergartens, economic shutdown, and limitations to healthcare access, welfare, and other support facilities. Following this first nationwide lockdown, periods with different levels of restrictive measures have alternated until summer 2021. Consequently, people, especially families, began facing new challenges (e.g., no out-of-home leisure activities, sharing limited space, working from home while taking care of children and/or home-schooling, and the absence of external support by other family members and social systems), which have been associated with a higher risk for the deterioration of health, quality of life, and intrafamilial relations [18,19,20,21,22]. Forced social distancing and home confinement enacted to contain the spread of the coronavirus produced alterations in life styles, greatly reduced leisure time, and changes in working conditions [23]. The confinement precipitated by the pandemic, as well as the almost complete loss of structured occupations (school, work, and training), have produced disturbances in all types of life activities in which people or groups participate [24,25,26]. Socioanagraphic (e.g., marital status, educational level, the size of one’s house, etc.) or behavioral variables (e.g., physical activity, hourly sleep patterns, eating habits, etc.) were identified as risks or protective factors related to psychological distress (especially in the dimensions of anxiety, stress, and depression) [24,27,28,29,30,31]. Although toddlers and children were not completely isolated since, in most cases, parents were at home and efforts were made by schools and teachers to maintain teaching activities and an active relationship with pupils, they have been exposed to drastic daily routine changes that have been particularly harmful for this demographic. The withdrawal from social life and daily activities (such as attending kindergarten/school) and the increased time spent on screens [32,33,34,35,36] combined with fear, anxiety, and the feeling of the unpredictable have increased the risk of developing distress and psychopathological symptoms or disorders [37] most acutely among older or socioeconomically disadvantaged children [38,39].

While there are several studies addressing the effects of the COVID-19 pandemic on children’s and adolescents’ mental health and family well-being [37,40,41], very little is known regarding the effects that changes in family routines, social life, and daily activities have had on children’s language and emotional–behavioral skills. Therefore, the aim of the present study was twofold. First, this study aimed to assess the long-lasting effects of changes in family and social life and in daily activities during the first nationwide lockdown (i.e., from March to June 2020) on children’s structural (i.e., speech, lexical, and grammatical) and pragmatic language skills and the emotional–behavioral profiles in a large sample of preschoolers aged 4 and 5 years living in Lombardy. Second, this study sought to analyze the moderation effects of children’s demographic variables (i.e., age and sex) and lifelong family characteristics (i.e., socioeconomic status, parental empowerment in managing everyday situations, exposure to reading, and number of siblings) on the long-lasting relationship between changes in family and social life and in daily activities and children’s language and emotional–behavioral skills during the first nationwide lockdown.

## 2. Materials and Methods

The study was conducted in accordance with the Declaration of Helsinki and the protocol was approved by the Ethical Committee of the Università Cattolica del Sacro Cuore (protocol number: 29–21, date of approval 6 April 2021).

### 2.1. Sample

Kindergartens belonging to the Federazione Italiana Scuole Materne (FISM) and located in Northern Italy (Lombardy region) were asked to participate in this study. Kindergartens that agreed to participate in this project invited caregivers of 4- and 5-year-old children to complete a web-based survey. Overall, 1238 surveys were collected. To ensure the consistency and appropriateness of the collected data, we removed surveys for which caregivers completed less than 85% of the items (N = 484) or surveys with a compiling time of less than 15 min (N = 17). In addition, we excluded surveys for which caregivers did not give their informed consent (N = 60). This led to 677 complete surveys (368 boys; age = 4.47 ± 0.59 years old). A total of 93% (N = 630) of the families declared that they had a perceived medium-low/medium-high income compared to the 2019 Italian average income [42]; 1.9% (N = 13) of families declared that they had a perceived low income; and 5.0% (N = 34) of families declared that they had a perceived high income.

#### 2.1.1. Web-Based Survey

A web-based survey, which took about 45 min to complete and was composed of 147 items, was developed on the Qualtrics platform [43]. The survey included both ad hoc items and subscales taken from validated questionnaires. Although the web-based survey had been available online since May 2021, most of the surveys (83.9%) were completed between November and December 2021.

##### Changes in Daily Life Related to the COVID-19 Pandemic

Information about changes in daily life related to the COVID-19 pandemic was derived from ad hoc questions. We collected retrospective information about who took care of a child (i.e., mother, father, grandparents, other family members, babysitter, or neighbors), the activities carried out by a child (i.e., watching TV, playing video games, engaging in leisure activities with family members, listening to songs/audiobooks, engaging in free play, and engaging in activities suggested by kindergarten staff), and parental employment (i.e., working from workplace, remote working, or unemployed due to layoffs, job loss, or other reasons for unemployment) during each lockdown period (i.e., from March to June 2020; from July to August 2020; from September 2020 to February 2021; and from March 2021 until the day the survey was completed). For both the persons who took care of a child and the activities carried out by the child, caregivers used a 5-point Likert scale (from never/less to always) to express the amount of time spent with the child or spent by the child, respectively. Regarding the activities carried out by the child during each lockdown period, we created two composite scores, namely, ‘TV-Video games’ and ‘Family activities’, as mean bivariate correlations were moderate among the items (data are available upon request). Finally, we collected information about the duration and frequency of quarantine periods; whether a family member or a close friend contracted SARS-CoV-2, the course of the disease, and the explanations given to the child; and distance teaching.

##### Demographic, Socio-Economic, and Obstetric Data

For each child, information about age, sex, gestational age, birth weight, attended class and type of school (i.e., public or private), and family environment (i.e., parental educational level and employment, spoken language, parental marital status, number of siblings, number of rooms in the child’s house, and area of residence) was collected. Parental employment was coded according to the Hollingshead’s 9-point scale [44]. Educational level was scored according to a 5-point ordinal scale based on the Italian school system (ranging between 10, corresponding to fifth-grade elementary school, and 90, equivalent to a post-doctoral degree) [45,46]. A comprehensive variable (Socio-Economic Status (SES)) was created by running a principal component analysis (PCA) concerning maternal and parental educational levels and employment (see Supplementary Materials).

##### Parental Empowerment

To measure parental empowerment in response to experiences, new conditions, or evolving circumstances in everyday situations, we used 12 items included in the ‘The family subscale’ of the self-reported Family Empowerment Scale (FES) [47,48]. A 5-point Likert scale (from 1 (never) to 5 (very often)) was used to grade the level of perceived empowerment for each item. Higher scores indicated higher levels of perceived empowerment. The sum of the scores of each item was used in further analysis.

##### Home Literacy Environment [49]

Information about the frequency with which parents read books to their child and encourage their child to write letters/words and how often the child looks at books and plays with pencils and/or crayons in an attempt to write was collected. Parents used an 8-point Likert scale (from 0 (never) to 7 (always)) to evaluate the frequency of the behavior described. The statements were grouped according to their area of investigation, i.e., exposure to reading (HLE_Read) and exposure to writing (HLE_Write). The mean scores of each area of investigation were computed and used in further analysis. Higher scores indicated a higher quality of a child’s HLE within each area.

##### Language Assessment

A short version of the *Children’s Communication Checklist—Second edition* (CCC-2) [50,51] was used to collect information about current language skills. The CCC-2 is a caregiver-rated questionnaire that quantifies the strengths and weaknesses of children’s communication using a 4-point numeric frequency scale (from 0 (less than once a week/never) to 3 (several times, i.e., more than twice, a day/always)). In this study, we used 35 items within five scales (Speech, “Does the child leave off beginning or ends of words?”; Syntax, “Does the child say things that sound babyish?”; Semantics, “Does the child mix up words of similar meanings?”; Initiation “Does the child talk repetitively about things no one else is interested in?”; and Context “Does the child miss the point of jokes or puns (though may understand slapstick humor)?”). Norms for Italian subjects are available, and z-scores corrected for age were used in further analysis. Three comprehensive variables (‘Structural Language’, ‘Initiation’, and ‘Context’) were created by running a PCA with respect to Speech, Syntax, Semantics, Initiation, and Context (see Supplementary Materials).

##### Emotional–Behavioral Assessment

Forty-five items of the caregiver-administered *Child Behavior Checklist for Ages 1.5–5* [52] were considered. In particular, we employed the DSM-Oriented scales (Affective, Anxiety, Pervasive Developmental, Attention Deficit/Hyperactivity (ADHD), and Oppositional Defiant). Parents were asked to answer questions concerning observed emotional–behavioral issues in the last two months. T-scores (mean = 50 ± 10) based on the normative sample of the Italian population [53,54] were used and entered as outcomes in subsequent analyses. Higher scores indicate greater problems.

### 2.2. Statistical Analysis

We considered demographic and lifelong variables (i.e., age, sex, SES, parental empowerment, HLE_Read, and number of siblings), activities carried out by the child during the COVID-19 pandemic’s first lockdown (i.e., TV–Video games, Family activities, and Kindergarten activities), language skills (Structural Language, Initiation, and Context), and emotional–behavioral traits (Affective, Anxiety, Pervasive Developmental, ADHD, and Oppositional Defiant). Descriptive statistics of and bivariate correlations among these variables were run in IBM SPSS Statistics for Windows, Version 28.0 [55].

**Figure 1:**
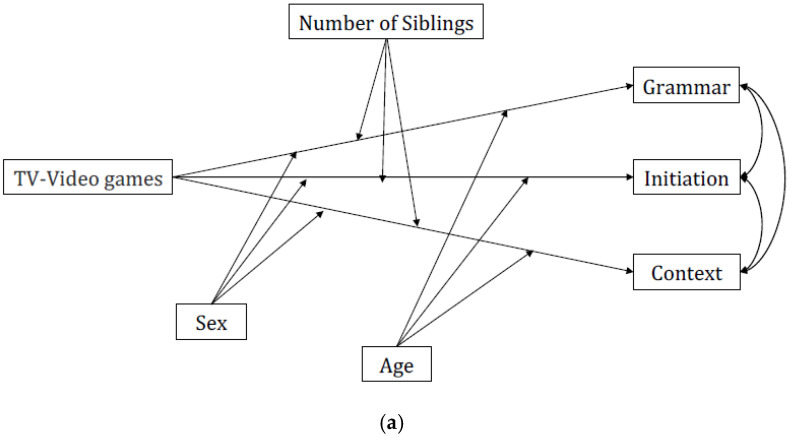

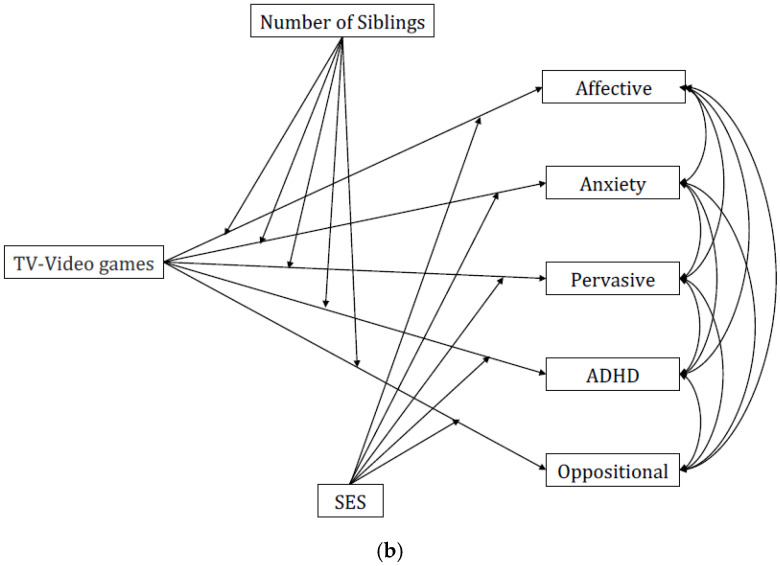
(**a**) The multiple-moderator model for language skills: TV–Video games = average score between watching TV and playing video games; language skills as defined by the PCA, including Speech, Syntax, Semantics, Initiation, and Context. (**b**) The multiple-moderator model for emotional–behavioral profiles: TV–Video games = average score between watching TV and playing video games; SES as defined by the PCA, including maternal and paternal educational level and employment.

Moderation effects were tested using Structural Equation Modeling as implemented in the Mplus 8.1 software package [56]. The chi-square statistic, the standardized root-mean-square residual (SRMR), the root-mean-square error of approximation (RMSEA), and the comparative fit index (CFI) were used to determine how adequately the data fit with the chosen models [57]. Moderation effects were checked for non-normality in terms of the product coefficient by applying the 5000-bootstrap assessment technique [56]. Confidence intervals of moderated associations not containing zero were indicators of significant moderation pathways [58,59]. A more stringent significance level (i.e., 99%) was applied to control for multiple testing effects. Based on the correlation matrix, two multiple-moderator models were tested: (1) the direct associations between activities carried out by the child during the COVID-19 pandemic’s first lockdown (i.e., TV–Video games) and language skills (Structural Language, Initiation, and Context) and the moderation effects of some of the lifelong variables (i.e., age, sex, and number of siblings) (Figure 1a), and (2) the direct associations between activities carried out by the child during the COVID-19 pandemic’s first lockdown (i.e., TV–Video games) and emotional–behavioral traits (Affective, Anxiety, Pervasive Developmental, ADHD, and Oppositional Defiant) and the moderation effects of some of the lifelong variables (i.e., SES and number of siblings) (Figure 1b). The full model’s findings were reported.

## 3. Results

Descriptive statistics of the included variables are reported in Table 1 and Table 2.

### 3.1. Bivariate Correlations between Lifelong Variables, COVID-19 Pandemic’s First Lockdown Variables, and Outcomes (Table 3)

#### 3.1.1. Lifelong Variables and Outcomes

A higher SES was significantly associated with lower emotional–behavioral traits (Affective problems *r* = −0.180, *p* < 0.001; Anxiety problems *r* = −0.091, *p* = 0.018; Pervasive developmental problems *r* = −0.123, *p* = 0.001; and ADHD problems *r* = −0.153, *p* < 0.001), better structural language and pragmatic skills (Grammar *r* = 0.199, *p* < 0.001, and Initiation *r* = 0.125, *p* = 0.001, respectively), and worse use of contextual information (Context *r* = −0.225, *p* < 0.001). Higher levels of parental empowerment were significantly associated with lower levels of emotional–behavioral problems (Affective problems *r* = −0.197, *p* < 0.001; Anxiety problems *r* = −0.201, *p* < 0.001; Pervasive developmental problems *r* = −0.189, *p* < 0.001; ADHD problems *r* = −0.235, *p* < 0.001; Oppositional problems *r* = −0.262, *p* < 0.001), better pragmatic skills (Initiation *r* = 0.179, *p* < 0.001) and worse use of contextual information (Context *r* = −0.166, *p* < 0.001). Higher HLE_Read was significantly associated with lower levels of affective and pervasive problems (Affective problems *r* = −0.096, *p* = 0.012; Pervasive developmental problems *r* = −0.139, *p* < 0.001), better structural language skills (Grammar *r* = 0.171, *p* < 0.001), and worse use of contextual information (Context *r* = −0.130, *p* = 0.001). An increasing number of siblings was significantly associated with fewer affective (*r* = −0.079, *p* = 0.040), anxiety (*r* = −0.106, *p* = 0.006), and ADHD problems (*r* = −0.111, *p* = 0.004).

#### 3.1.2. Variables and Outcomes Related to First Lockdown of the COVID-19 Pandemic

Spending more time watching TV/playing video games was significantly associated with higher levels of affective (*r* = 0.151, *p* < 0.001) and anxiety (*r* = 0.088, *p* = 0.021) problems, better use of contextual information (*r* = 0.108, *p* = 0.005), and worse structural language (*r* = −0.166, *p* < 0.001) and pragmatic (*r* = −0.139, *p* < 0.001) skills. Spending more time engaging in family activities and kindergarten activities was significantly associated with lower levels of oppositional defiant problems (*r* = −0.090, *p* = 0.020 and *r* = −0.077, *p* = 0.046, respectively).

#### 3.1.3. Lifelong Variables and Those Related to First Lockdown of the COVID-19 Pandemic

A higher SES was significantly associated with less time spent watching TV/playing video games (*r* = −0.256, *p* < 0.001) and on activities proposed by a kindergarten/family (*r* = −0.169, *p* < 0.001 and *r* = −0.136, *p* < 0.001, respectively). A higher level of parental empowerment was significantly associated with increased time spent engaging in activities proposed by a kindergarten/family (*r* = 0.096, *p* = 0.013 and *r* = 0.118, *p* = 0.002, respectively) and reduced time spent watching TV/playing video games (*r* = −0.079, *p* = 0.039). A higher level of HLE_Read was significantly associated with increased time spent on family activities (*r* = −0.103, *p* = 0.007) and reduced time spent watching TV/playing video games (*r* = −0.257, *p* < 0.001).

### 3.2. Moderation Effects—The Multiple-Moderator Models

#### 3.2.1. Language Skills (Figure 1a)

The multiple-moderator model accounted for approximately 0.5%, 4.5%, and 5.3% of the variance of Structural Language, Initiation, and Context, respectively. Using 99% confidence intervals and following the performance of 5000 bootstrapping analyses, no significant moderation effects were found. However, a trend toward significance was found for the interaction between LD_VideoTV and number of siblings with respect to Initiation (Standardized β = 0.398, Standard Error (SE) = 0.073, and 95% CI = 0.032/0.314). According to the simple slopes test, the negative association between ‘LD_VideoTV’ and ‘Initiation’ was significant for only children (β = −0.369, SE = 0.090, t = −4.121, and 95% CI = −0.544/−0.193) but not for children with siblings (β = −0.100, SE = 0.064, t = −1.567, and 95% CI = −0.225/0.025).

#### 3.2.2. Emotional–Behavioral Traits (Figure 1b)

The multiple-moderator model accounted for approximately 6.6%, 2.5%, 2.3%, 11.4%, and 9.4% of the variance of the Affective, Anxiety, Pervasive Developmental, ADHD, and Oppositional Defiant traits, respectively. Using 99% confidence intervals and 5000 bootstrapping analyses, a significant moderation effect was found. In particular, the relationships between LD_VideoTV and Affective problems was moderated by the number of siblings (Standardized β = −0.558, SE = 0.372, and 99% CI = −2.486/−0.561). According to the simple slopes test, the positive association between LD_VideoTV and Affective problems was significant for only children (β = 3.184, SE = 0.503, t = 6.337, and 95% CI = 2.197/4.1971) but not for children with siblings (β = 0.148, SE = 0.358, t = 0.412, and 95% CI = −0.556/0.851). Moreover, a trend toward significance was found for the interaction between LD_VideoTV and number of siblings with respect to Oppositional Defiant problems (Standardized β = −0.515, SE = 0.668, and 95% CI = −2.710/−0.165). However, the simple slopes analysis did not return a significant *p*-value, meaning that the two slopes (i.e., only children versus children with siblings), although being significantly different from one another, were not significantly different from zero [60].

## 4. Discussion

This study builds on previous results demonstrating the effects of the quality and quantity of environmental inputs/stimuli on the development of children’s cognitive, linguistic, and emotional–behavioral skills [1]. Taken together, our findings show that higher scores in lifelong variables (i.e., SES, parental empowerment in managing everyday situations, exposure to reading, and number of siblings) and spending less time watching television and/or playing video games were significantly associated with fewer emotional–behavioral problems and better structural language and pragmatic skills. These data support previous evidence describing a higher level of parental education and family SES as well as a stimulating HLE and having siblings as important protective resources in terms of children’s cognitive, linguistic, and emotional–behavioral development [61,62,63,64,65,66]. On the contrary, the correlations between pragmatic skills involved in the use of contextual information and both lifelong variables and the activities carried out by a child during the COVID-19 pandemic’s first lockdown showed an unexpected pattern. In particular, as recent studies showed that children’s pragmatic skills are related to the quality and quantity of socio–cognitive interactions [67], we would have expected a negative correlation between lockdown variables and Context. Moreover, although much less is known about the correlation between lifelong variables and the specific skills involved in using contextual information, we would have expected a positive correlation between these variables. Previous studies reported no correlations between pragmatic skills and parental education and income [67]. Nonetheless, we hypothesized that parents with a higher socio-economic and educational background may have higher expectations of their children’s pragmatic abilities and, therefore, might be more critical when judging them. Alternatively, it is plausible that these parents are more prone to detect children’s subtle pragmatic difficulties concerning the correct use of contextual information and the distinction between literal and figurative language. However, we cannot determine whether the difficulties reported by the parents are real (and thus correctly judged) or only perceived (potentially deriving from excessive expectations) as we did not collect objective measures of the children’s language abilities. Furthermore, we cannot establish whether this negative correlation was better explained by the special conditions characterizing the first COVID-19-related lockdown as opposed to a stable phenomenon.

More interestingly, additional significant moderating effects were found. In particular, the interaction between the amount of time spent watching TV/playing video games and the presence of siblings significantly accounted for long-term emotional profiles and explained about 7% of the variance in children’s affective problems. These findings suggested that a portion of the previously described negative effects conferred by watching TV/playing video games on affective profiles in preschoolers [68,69] is attenuated by the presence of siblings. Previous studies reported controversial findings about the effect of the use of screen-based technology on emotional–behavioral outcomes during the pandemic [36,70]. The moderating effect of having siblings on the negative consequences of TV time on emotional adjustment could be interpreted in the perspective of shared (active) vs. non-shared (passive) screen time. As thoroughly explained by some studies [71,72,73], co-viewing offers parents an opportunity to focus on interacting sensitively with their child and actively verbalizing, scaffolding, and discussing the content on the screen. It could be assumed that the presence of siblings acts in a similar way. The only child status has emerged as a risk factor for cognitive and socio-emotional development [74,75,76,77]. Previous studies have revealed that only children exhibit more positive developmental outcomes, more positive relationships with their parents, and fewer behavioral problems in school compared with non-only-children [75,78]. Otherwise, only children receive too much attention and excessive praise from their parents and grandparents [79], which may foster undesirable personality traits [78,79]. Additionally, due to the absence of siblings, only children miss many opportunities to develop/foster social and interpersonal skills, emotional support, and learning opportunities compared with non-only children [64,66,80].

Notwithstanding the novelty of the present results, they need to be considered within the limitations of the study. First, we asked parents to retrospectively report information about changes in daily life related to the COVID-19 pandemic. Although this increases the probability of recall bias, it should also be considered that we collected actual information regarding an emotionally relevant period. The memory of emotionally salient events is less subject to recall bias [81]. On the other hand, information about children’s language skills and emotional–behavioral profiles was not collected retrospectively since we asked parents to report their children’s current skills. Second, we asked caregivers to complete questionnaires about their child’s language skills and emotional–behavioral profiles. Bias associated with self-report questionnaires is quite common and can potentially influence outcomes. However, they are widely used as proxy measures of outcomes [82]. Third, 93% of our sample had a perceived medium level of income. As SES has a significant impact on the outcomes measured in this study, our findings cannot be generalized to the whole Italian population. However, these results provide important information about the influence of specific protective factors during the COVID-19 pandemic.

## 5. Conclusions

Taken together, our data show how the long-lasting effects of changes in family and social life and in daily activities during the first COVID-19-pandemic-related lockdown on a child’s linguistic and emotional–behavioral profiles are moderated by lifelong family characteristics. An only child status represented a risk factor moderating the effects of the lockdown-related limitations on socio-emotional development. This had already been a well-established risk factor ahead of the pandemic. Thus, children who could be at high risk in more normal circumstances may have been more acutely impacted by the pandemic. Therefore, assessing the long-term effects of lockdown-related measures and how these could have been moderated by potential risk/protective factors adds significant information to the existing literature.

## Figures and Tables

**Table 1:** Descriptive statistics of lifelong variables, emotional and behavioral traits, and language skills in the total sample (*n* = 677).

		Min	Max	Mean	Standard Deviation	Skewness	Kurtosis
**Lifelong variables**	**Education Mother**	20	80	58.71	15.7	−0.82	0.38
	**Education Father**	15	80	50.56	17.83	−0.47	−0.6
	**Occupation Mother**	0	80	57.9	16.78	0.08	−0.8
	**Occupation Father**	20	80	56.15	15.11	0.21	−0.42
	**Number of Siblings**	0	5	0.77	0.69	0.82	1.93
	**FES**	2.36	4	3.55	0.34	−0.64	−0.24
	**HLE_Read**	0	14	11.77	2.88	−1.76	2.77
**Emotional and** **behavioral traits**	**Affective Problems**	50	76	54.45	5.73	1.56	1.69
	**Anxiety Problems**	50	86	55.73	7.07	1.43	1.8
	**Pervasive Developmental Problems**	50	86	55.51	6.88	1.44	1.47
	**ADHD Problems**	50	76	53.98	4.95	1.64	2.45
	**Oppositional Problems**	50	80	52.53	4.5	2.62	7.56
**Language skills**	**Speech**	−4.71	0.82	−0.16	1.07	−1.54	1.92
	**Syntax**	−8.7	0.81	−0.13	1.31	−2.48	7.94
	**Semantics**	−4.32	1.77	−0.36	0.98	−0.51	0.72
	**Initiation**	−3.94	2.36	−0.91	1.04	−0.26	0.11
	**Context**	−3.85	2.18	−0.29	1.1	−0.37	0.05

Education was coded according to a 5-point scale (between 10 and 90) based on the Italian school system [45,46]; occupation was coded according to the Hollingshead’s scale (between 0 and 90) [44]; FES = Family Empowerment Scale; HLE_Read = Home Literacy.

**Table 2:** Descriptive statistics of activities carried out by the child during the COVID-19 pandemic’s first lockdown in the total sample (*n* = 677).

			Amount of Time Spent By the Child				
			Never or Less	25%	50%	75%	100%
**Activities carried out by the child during the** **COVID-19 pandemic’s first lockdown**	**Kindergarten Activities**	**Frequency**	376	160	69	33	39
		**%**	55.5	23.6	10.2	4.9	5.8
	**Watching TV**	**Frequency**	92	301	205	63	16
		**%**	13.6	44.5	30.3	9.3	2.4
	**Playing video games**	**Frequency**	414	169	70	19	5
		**%**	61.2	25	10.3	2.8	0.7
	**Leisure activities with** **family members**	**Frequency**	12	142	279	182	62
		**%**	1.8	21	41.2	26.9	9.2
	**Free play**	**Frequency**	7	111	204	194	161
		**%**	1	16.4	30.1	28.7	23.8
	**Listening to songs or** **audiobooks**	**Frequency**	176	256	144	71	30
		**%**	26	37.8	21.3	10.5	4.4

**Table 3:** Correlations between lifelong variables, activities during the COVID-19 pandemic’s first lockdown, emotional/behavioral traits, and language skills.

		Lifelong Variables			COVID-19 Pandemic’s First Lockdown			Emotional–Behavioral Traits					Language Skills		
		FES	HLE_Read	Number of Siblings	Kindergarten Activities	TV–Video Games	Family Activities	Affective Problems	Anxiety Problems	Pervasive Developmental Problems	ADHD Problems	Oppositional Problems	Grammar	Initiation	Context
**Lifelong variables**	**SES**	0.118 **	0.264 **	0.086 *	−0.169 **	−0.256 **	−0.136 **	−0.180 **	−0.091 *	−0.123 **	−0.153 **	−0.017	0.199 **	0.125 **	−0.225 **
	**FES**		0.052	−0.053	0.096 *	−0.079 *	0.118 **	−0.197 **	−0.201 **	−0.189 **	−0.235 **	−0.262 **	0.045	0.179 **	−0.166 **
	**HLE_Read**			0.024	−0.010	−0.257 **	0.103 **	−0.096 *	−0.074	−0.139 **	−0.064	0.030	0.171 **	0.002	−0.130 **
	**Number of** **siblings**				0.021	−0.002	−0.002	−0.079 *	−0.106 **	−0.073	−0.111 **	−0.048	−0.005	0.057	−0.001
**COVID-19 pandemic’s first locdown**	**Kindergaten Activities**					0.194 **	0.272 **	0.023	−0.032	−0.065	−0.021	−0.090 *	−0.031	−0.015	0.048
	**TV–Video** **games**						0.213 **	0.151 **	0.088 *	0.043	0.073	0.041	−0.166 **	−0.139 **	0.108 **
	**Family** **activities**							0.011	−0.045	−0.070	−0.069	−0.077 *	0.030	−0.073	0.015

Bivariate Pearson’s correlations in the total sample (N = 677). * Two-tailed *p*-value < 0.05; ** Two-tailed *p*-value < 0.01. SES as defined by the PCA including maternal and paternal educational levels and employment; FES = Family Empowerment Scale; HLE_Read = Home Literacy Environment, concerning exposure to reading; TV–Video games = average score between watching TV and playing video games; Family activities = average scores among leisure activities with family members, listening to songs/audiobooks, and free play; language skills as defined by the PCA, including Speech, Syntax, Semantics, Initiation, and Context.

## Data Availability

The data presented in this study are available on request from the corresponding author. The data are not publicly available due to ethical restrictions.

## Supplementary Materials

The following supporting information can be downloaded at https://www.mdpi.com/article/10.3390/children10040656/s1, Table S1: Rotated component matrix concerning maternal and parental educational levels and employment (extraction method: principal component analysis; rotation method: oblimin); Table S2: Rotated component matrix concerning Speech, Syntax, Semantics, Initiation, and Context (extraction method: principal component analysis; rotation method: oblimin); File S1: Supporting Materials and Methods [44,83].
