# Supplementary material for: Long-Lasting Effects of Changes in Daily Routine during the Pandemic-Related Lockdown on Preschoolers’ Language and Emotional–Behavioral Development: A Moderation Analysis
Source: Children (Basel). 2023 Mar 30;10(4):656. doi: 10.3390/children10040656 (PMC10137118; doi:10.3390/children10040656)
Supplement: Supplementary file 1 [file children-10-00656-s001.zip › Revised_Suppl Materials_Children_21mars2023.pdf]

Materials and Methods

Ad-hoc web-based survey

Demographic, socio-economic and obstetric data

In order to create a comprehensive variable and according to the correlations between educational level and parental employment (mean bivariate correlations (r)=0.364; data available upon request), we ran a principal component analysis by including mother’s educational level and employment, and father’s educational level and employment, to find the optimal weights for the variables to account for the maximum amount of variance in the dataset with the smallest number of underlying factors [1]. Using an oblimin rotation method, we obtained one factor with an eigenvalue >1.0, i.e., ‘Socio-Economic Status’ (SES), explaining 52.35% of the total variance (Kaiser-Meyer-Olkin measure of sample adequacy=0.596, Bartlett test of sphericity, X^2=562.583, df=6, p<0.001; Supplementary Table 1). Standardized regression scores have been saved for each subject.

Language assessment

As mean correlations were moderate among these scales (r=0.333; data available upon request), we ran a principal component analysis [1]. Using an oblimin rotation method, we obtained three factors, i.e., ‘Structural Language’, ‘Initiation’ and ‘Context’, explaining 47.76%, 19.74% and 12.90% of the total variance, respectively (Kaiser-Meyer-Olkin measure of sample adequacy=0.751, Bartlett test of sphericity, X2=672.187, df=10, p<0.001; Supplementary Table 2). Standardized regression scores were saved for each subject and entered as outcomes in subsequent analyses.

References

[1] Norman GR, Streiner DL (2008) Analysis of variance. In: Biostatistics: The Bare Essentials, 3rd ed.; BC Decker: Hamilton, ON, Canada, pp 77-80
